# Consecutive antibiotic use in the outpatient setting: an extensive, longitudinal descriptive analysis of antibiotic dispensing data in the Netherlands

**DOI:** 10.1186/s12879-019-3732-x

**Published:** 2019-01-24

**Authors:** Loek A. W. de Jong, Paul D. van der Linden, Monique M. B. Roukens, Ewoudt M. W. van de Garde, Alike W. van der Velden, Stephanie Natsch, on behalf of SWAB’s Working Group on Surveillance of Antimicrobial Use

**Affiliations:** 1 Department of Pharmacy, Radboud University Medical Center, Radboud Institute for Health Sciences, P.O. Box 9101, 6500 HB Nijmegen, the Netherlands; 2 Department of Clinical Pharmacy, Tergooi Hospital, van Riebeeckweg 212, 1213 XZ Hilversum, Netherlands; 3 Department of Pharmaceutical Science, Division of Pharmacoepidemiology and Clinical Pharmacology, Utrecht University, Universiteitsweg 99, 3584 CA Utrecht, the Netherlands; 4 Julius Center for Health Sciences and Primary Care, University Medical Center Utrecht (STR 6.103), Heidelberglaan 100, 3584 CX Utrecht, the Netherlands

**Keywords:** Antibacterials, Antibiotic prescription, Antibiotic usage, Antibiotics, Consecutive antibiotic use, Outpatient setting, Switch, Antibiotic failure, Treatment failure

## Abstract

**Introduction:**

Taking consecutive antibiotic use into account is of importance to obtain insight in treatment within disease episodes, use of 2nd- and 3rd-choice antibiotics, therapy failure and/or side effects. Nevertheless, studies dealing with consecutive antibiotic use are scarce. We aimed at evaluating switch patterns in antibiotic use in the outpatient setting in the Netherlands.

**Methods:**

Outpatient antibiotic dispensing data was processed to antibiotic treatment episodes consisting of single prescriptions or consecutive prescriptions (2006 to 2014). Consecutive prescriptions were categorised into prolongations and switches. Switches were further analysed to obtain antibiotic switch percentages and trends over time. Outcomes were compared with recommendations of Dutch guidelines.

**Results:**

A total of 43,179,867 antibiotic prescriptions were included in the analysis, consisting of single prescriptions (95%), prolongations (2%) and switches (3%). The highest switch percentages were found for trimethoprim (7.6%) and nitrofurantoin (5.4%). For fosfomycin, ciprofloxacin, flucloxacillin and trimethoprim we found the highest yearly increase in switching. Amoxicillin/clavulanic acid was most often used as second antibiotic in a switch. A surprisingly high number of 2nd- and 3rd-choice antibiotics are prescribed as first antibiotic in a treatment.

**Conclusions:**

Although the actual reason for a switch is unknown, switch patterns can reveal problems concerning treatment failure and guideline adherence. In general, switch percentages of antibiotics in the Netherlands are low. The data contributes to the knowledge regarding antibiotic switch patterns in the outpatient setting.

## Introduction

The medical impact and economic burden of antibiotic resistance is tremendous and increasing annually [1]. Antibiotic resistance is a direct result of antibiotic (mis)use and therefore appropriate use of antibiotics is of utmost importance [2]. Approximately 80–90% of total human antibiotic consumption occurs in the outpatient setting [3–5]. Collecting data on outpatient antibiotic use is essential for adjusting practice to ensure appropriate use of antibiotics. Published reports concerning antimicrobial drug use and resistance offer insight in trends and, therefore, play a major role in policymaking [3–7].

A wide variety of indicators exist in literature to quantify and qualify antimicrobial use, without consensus on the most appropriate indicators to be used [8–10]. Overall, quality indicator analyses consider each antibiotic prescription as an individual course and do not take consecutive antibiotic use into account. Patients might need more than one prescription during an infectious disease episode. The treatment strategy can change, or be extended due to inadequate symptom resolution, results of antimicrobial susceptibility tests, allergies, adverse drug reactions, therapeutic noncompliance, or as a result of an initially wrong diagnosis.

To retrieve reliable results on consecutive antibiotic use, the index date of the antibiotic and the duration of the therapy have to be well documented and a unique patient identifier is needed. Therefore only databases containing high quality prescription or dispensing data are suitable for the analysis of consecutive antibiotic use in the outpatient setting.

So far, only a few studies deal with consecutive antibiotic use [11–14]. These studies focus on specific indications [11, 12], individual antibiotics [13], or specific patient groups [14]. The main outcome in these studies is treatment failure, defined as a refill of the index antibiotic dispensed after the completed days of therapy (prolongation), a prescription of a different antibiotic within one month after the index prescription (switch), hospitalization, or a visit to the emergency department. It has been shown that the vast majority of treatment failures include antibiotic switches [11, 12].

Besides calculating treatment failure for individual antibiotics, it is of great interest to visualize antibiotic switches and focus on second prescriptions within an antibiotic treatment. It is of importance whether 2nd/3rd choice antibiotics are prescribed as first one, which is often inappropriate, or as second one within an infectious disease episode, which could be appropriate for severe infections, or to treat pathogens resistant to the 1st choice antibiotic. Therefore, studies dealing with consecutive antibiotic use can provide valuable insight. We aimed at evaluating switch patterns in antibiotic use in the outpatient setting in the Netherlands.

## Methods

### Data collection

Data on outpatient antibiotic use in the Netherlands was obtained from the Foundation for Pharmaceutical Statistics (SFK) for the period 2006–2014. The SFK collects dispensing data from participating Dutch community pharmacies. The participation rate increased from 90% in 2006 to 95% in 2014. The dataset contained a pharmacy number, a unique patient number, the anatomical therapeutic chemical classification code (ATC-5) of the drug [15], the start date, prescribed dose and the number of dispensed units. In the Netherlands, the exact number of drug units to complete the prescribed course is dispensed at the pharmacy.

### Study population

The study population consists of all patients who filled a prescription for oral antibiotics (ATC J01) at a SFK participating Dutch community pharmacy from January 1, 2006 to December 31, 2014. As the SFK covers 95% of pharmacies, the study population is a proper representative of the Dutch population.

### Data handling and classification

SFK data, provided in Microsoft Office Excel® 2007, was processed to SPSS statistics 22 and organised per year. Only antibiotics for systemic use (ATC-code J01) were included. All antibiotic prescriptions were sorted by patient number. A stop date for each prescription was calculated using the start date, the number of dispensed units and the prescribed dose, resulting in the duration of the course. The total duration of antibiotic therapy for all patients was calculated for each successive year. The antibiotic treatment episodes were composed of single prescriptions and consecutive prescriptions. A consecutive prescription was defined as a successive antibiotic prescription for the same patient within the period from prescribing the first antibiotic up to 3 days after the calculated end date of the first antibiotic prescription. Consecutive prescriptions were further categorised as prolongation, or switch. A prolongation was defined as a repeat prescription of the same antibiotic, and a switch was defined as a consecutive prescription with another antibiotic.

**Fig. 1:**
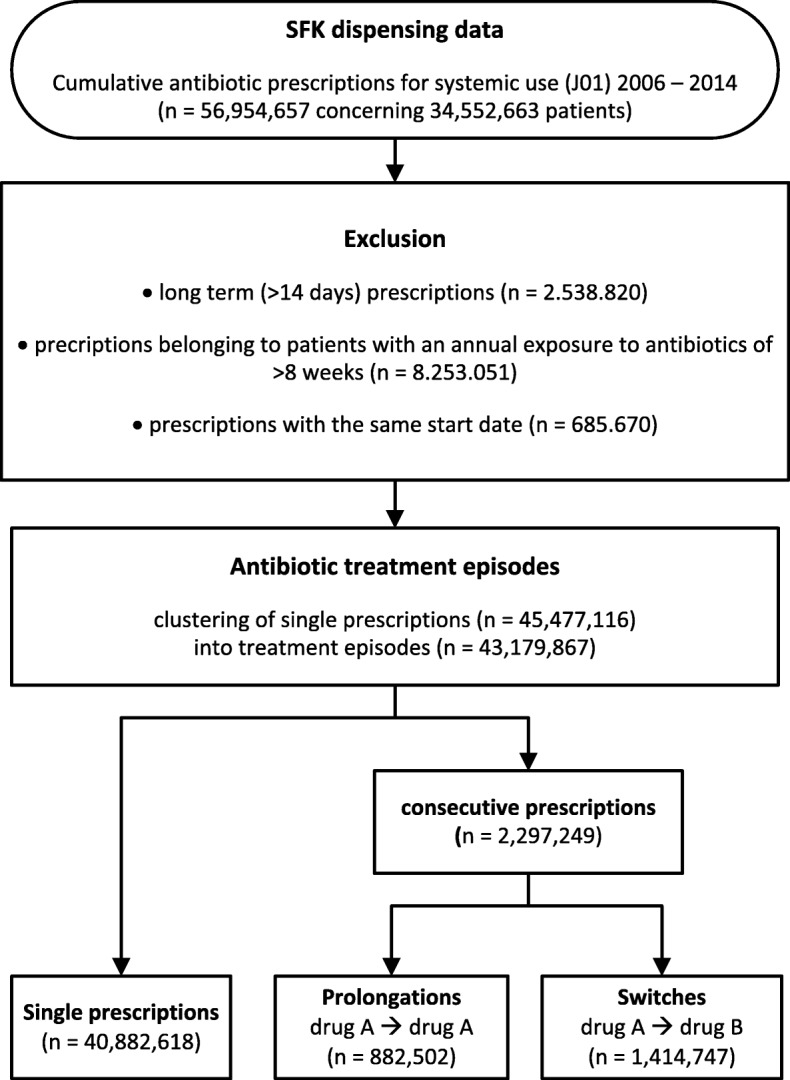
Overview of data handling

Prescriptions for one patient with the same start date, single antibiotic prescriptions with a duration > 14 days and patients with an annual exposure to antibiotics of more than 8 weeks were excluded (Fig. 1).

### Data analyses

Analysis of the data was performed using Microsoft Office Excel® 2007 and SPSS statistics 22. We first counted the total number of single prescription-, prolongation- and switched treatment episodes per year. Next we calculated the percentage of prolongations and switches by dividing the number of prolongations and switches by the total number of treatment episodes. Data were presented for the nine most often prescribed antibiotics, representing > 90% of all antibiotic prescriptions. The other antibiotics were grouped as others. The yearly switch percentage for each antibiotic was calculated and plotted in a graph to display trends over time. The yearly distribution of secondly used antibiotics in a switch were displayed in a stacked clustered column chart. A Sankey diagram was created using the open source web tool RAWGraphs® [16] to visualise the most common antibiotic switches in the year 2014.

### Statistical analyses

Statistics were conducted with IBM SPSS Statistics 22. For each year from 2006 to 2014, we determined antibiotic switch rates for the nine most often prescribed antibiotics. Generalised linear models with a binomial family and an identity link were used to evaluate whether the linear trends were statistically significant. To assess an association between year and the distribution of second used antibiotics we performed a chi-square test.

## Results

The vast majority of antibiotic treatments concerned single prescriptions (95%). Numbers and distribution of antibiotic treatment episodes per year are shown in Table 1. Antibiotic treatment episodes consisted for 2% of prolongations and for 3% of switches.

**Table 1:** Total number of single prescriptions, prolongations and switches per year

	2006	2007	2008	2009	2010	2011	2012	2013	2014	total	Percentage^a^
single prescriptions	4,019,710	4,421,525	4,716,882	4,834,467	4,739,070	4,552,601	4,668,455	4,546,357	4,383,551	40,882,618	95%
prolongations	88,138	94,759	99,839	104,351	104,148	98,446	100,340	97,282	95,199	882,502	3%
switches	141,214	153,475	158,186	164,756	164,307	159,535	165,845	154,862	152,567	1,414,747	2%
total	4,249,062	4,669,759	4,974,907	5,103,574	5,007,525	4,810,582	4,934,640	4,798,501	4,631,317	43,179,867	100%

^a^ Percentages were stable from 2006 to 2014

The distribution of single prescriptions, prolongations and switches for the nine most common used antibiotics is shown in Table 2 for the year 2014. This table also shows the number of antibiotics that were used as second in a switch.

**Table 2:** Distribution of first and second used antibiotics in the year 2014

Antibiotic agent	Single prescriptions *n* (% of total)	Prolongations *n* (% of total)	First used in a switch (drug A)^a^ *n* (% of total)	Second used in a switch (drug B)^a^ *n* (% of total)
amoxicillin	1,044,483 (24%)	18,924 (20%)	27,376 (18%)	12,264 (8%)
nitrofurantoin	717,571 (16%)	7922 (8%)	41,311 (27%)	8264 (5%)
amoxicillin/clavulanic acid	622,430 (14%)	22,259 (23%)	17,370 (11%)	33,862 (22%)
doxycycline	587,598 (13%)	8735 (9%)	17,699 (12%)	11,333 (7%)
azithromycin and clarithromycin	412,609 (9%)	7561 (8%)	9438 (6%)	24,079 (16%)
flucloxacillin	263,599 (6%)	13,871 (15%)	9931 (7%)	3515 (2%)
ciprofloxacin	233,127 (5%)	6168 (6%)	8131 (5%)	19,968 (13%)
pheneticillin	134,071 (3%)	1890 (2%)	5530 (4%)	991 (1%)
trimethoprim	72,794 (2%)	987 (1%)	6084 (4%)	8729 (6%)
fosfomycin	87,252 (2%)	844 (1%)	3129 (2%)	13,203 (9%)
others	208,017 (5%)^b^	6038 (6%)^c^	6568 (4%)^d^	16,359 (11%)^e^
total	4,383,551 (100%)	95,199 (100%)	152,567 (100%)	152,567 (100%)

^a^ Switches: drug A ➔ drug B

^b^ The group others consist of antibiotics representing ≤1% of total single prescriptions

^c^ The group others largely consist of clindamycin, representing 2% of the total prolongations. All others represent ≤1% of total prolongations

^d^ The group others consist of antibiotics representing ≤1% of the first ones used in a switch

^e^ The group others largely consist of sulfamethoxazole/trimethoprim, clindamycin and norfloxacin, representing 3, 2 and 2% of the second ones used in a switch. All other antibiotics represent ≤1% of the second ones used in a switch

**Fig. 2:**
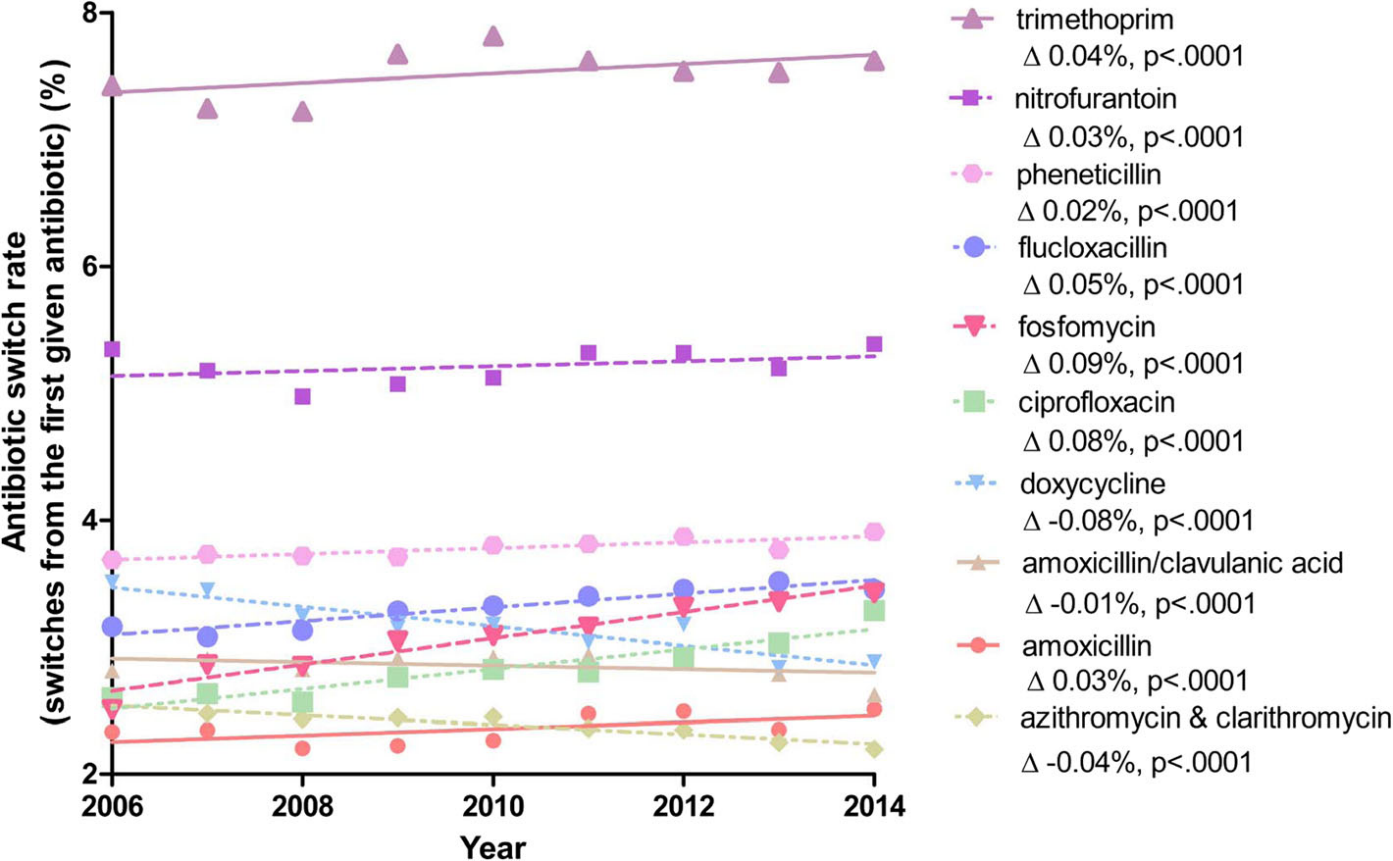
Analysis of antibiotic switch rate time trends using generalised linear models

Trimethoprim and nitrofurantoin showed relatively high switch percentages of 7.6 and 5.4% in 2014. In other words, a high percentage of initial prescriptions of these drugs were followed by a second antibiotic prescription. The switch percentages for the other antibiotics were between 2 and 4%. The time-trend from 2006 to 2014 in yearly switch percentages (from the first given antibiotic) is shown in Fig. 2. Differences in switch percentages over this period were minimal. The highest yearly increase in switch percentage was found for fosfomycin (0.09%/year), ciprofloxacin (0.08%/year), flucloxacillin (0.05%/year) and trimethoprim (0.04%/year). On the other hand, switching to a second antibiotic after initial use of doxycycline (− 0.08%/year), azithromycin and clarithromycin (− 0.04%/year) and sulfamethoxazole/trimethoprim (− 0.04%/year) declined over time [data not shown].

**Fig. 3:**
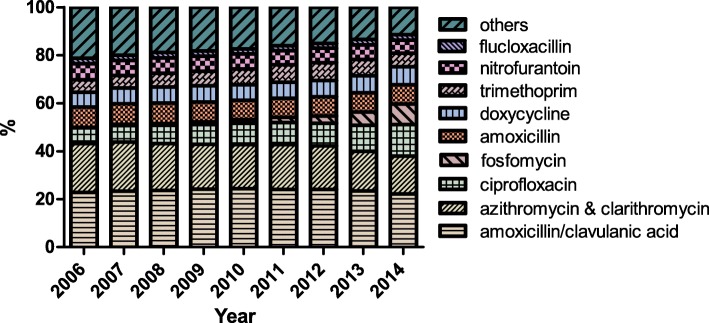
Time-trends in distribution of second used antibiotics in a switch. Pearson Chi-square test confirmed a relation between year and distribution, *p* < .0001

Figure 3 shows time-trends in the distribution of the second antibiotic used in a switch. Amoxicillin/clavulanic acid was most often used as second antibiotic in a switch. This finding is consistent over time. A clear increase was seen for switches in which fosfomycin and ciprofloxacin were used as second antibiotic.

**Fig. 4:**
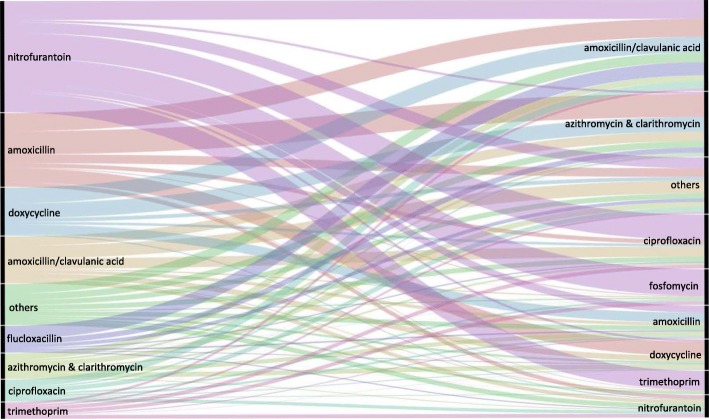
Sankey diagram of all antibiotic switches in the year 2014. Left side: the top-9 initial antibiotics that are followed by a second prescription within an antibiotic treatment episode. Right side: the top-9 antibiotics that are used as second prescription in a switch. The group others on the left side of the figure reflects 10% of the total switches, mainly consisting of pheneticillin (36%), fosfomycin (21%) and cotrimoxazole (14%). The group others on the right side of the figure reflects 14% of second prescriptions in a switch. This group predominantly involves cotrimoxazole (24%), flucloxacillin (17%) clindamycin (16%) and norfloxacin (14%)

Figure 4 is an overview of all switches (*n* = 1,414,747) in the year 2014. In this figure the most common switch patterns can be seen at a glance. For nitrofurantoin these concerned switches to fosfomycin (24%), ciprofloxacin (23%), trimethoprim (17%) or amoxicillin/clavulanic acid (17%). Switches after first use of amoxicillin most often go to one of the macrolides, azithromycin and clarithromycin (31%), amoxicillin/clavulanic acid (25%) or doxycycline (19%).

## Discussion

### Principal findings

To the best of our knowledge, this is the first study giving an overview of all first and second prescriptions within antibiotic treatment episodes from a national database of outpatient antibiotic use. The vast majority of antibiotics, including antibiotics that are generally considered as second- or third-choice treatments, are prescribed as first antibiotic in a treatment. This finding indicates that the initial choice of the prescriber is not always in line with recommendations in guidelines.

### Comparison with literature

Previously conducted studies regarding consecutive antibiotic use report high treatment failure rates [11, 12]. A study performed in the United States of America reports unadjusted failure rates ranging from 20 to 24% for antibiotics prescribed for treatment of community-acquired pneumonia [11]. A study from the United Kingdom reports an overall treatment failure rate of 15% for antibiotics prescribed for the treatment of upper and lower respiratory tract infections, skin and soft tissue infections, and acute otitis media [12]. The vast majority of treatment failures include antibiotic switches as is demonstrated in the study of Currie et al. reporting a switch in 94% of failures [12]. A recent study from Norway reports an average switch rate of 6% for doxycycline, amoxicillin, phenoxymethylpenicillin and macrolides [13]. A Danish study evaluated prescribing patterns of antibiotics among Danish children and found switch percentages of 5 and 1% for phenoxymethylpenicillin and amoxicillin [14]. Although we cannot directly compare our results with these data, due to different settings and studies, differences in switch rates between countries is apparent. Switch rates in the Netherlands seems to be low in comparison with US, UK and Norway.

We found the highest switch percentages for trimethoprim and nitrofurantoin. These antibiotics are predominantly used to treat urinary tract infections and the switch percentages indicate that initial treatment with these antibiotics is quite often followed by another antibiotic. According to the Dutch national guidelines, nitrofurantoin is the first choice treatment for uncomplicated urinary tract infections in the outpatient setting [17]. Fosfomycin should be used as second choice and trimethoprim as third [17]. The total use of nitrofurantoin significantly increased from 1.0 defined daily dose (DDD) /1000 inhabitant-days to 1.4 DDD/1000 inhabitant-days [5], and the switch percentage for nitrofurantoin showed a minor yearly increase. Trimethoprim showed a relatively high switch percentage of 7.6%. This might be explained by high resistance to trimethoprim among urine-isolated *E. coli*, K. *pneumonia* and *P. mirabilis*, the predominant species causing urinary tract infections in the Netherlands [5]. It was shown that fosfomycin, trimethoprim and ciprofloxacin are often used as second prescription after initial treatment with nitrofurantoin. This is in line with the Dutch guidelines for the treatment of urinary infections [17]. However, the vast majority of prescriptions for fosfomycin, trimethoprim and ciprofloxacin concern first prescriptions in a treatment, which is not in line with guidelines. A decrease in the switch percentage for sulfamethoxazole/trimethoprim is in line with a decreasing resistance for this agent in the primary care setting in the Netherlands [5]. In the Netherlands, presentation of urological indications has been increasing, with 148 episodes per 1000 patient-years in 2010; the prescribing rate for urological indications remained about 50% [18].

For ciprofloxacin we found a yearly increase in total use and a corresponding annual increase of 0.08% in its switch percentage. This is worrisome as it could reflect increased bacterial resistance to ciprofloxacin. Ciprofloxacin often is a 3rd choice treatment in Dutch primary care and reserved for severe infections and hospitalised patients, and resistance in primary care jeopardises this aim.

Not surprisingly, amoxicillin/clavulanic acid is the drug that is most often used as second antibiotic in a switch. It is used after a wide variety of first prescriptions, including nitrofurantoin, amoxicillin, doxycycline, flucloxacillin, a macrolide and ciprofloxacin. A second prescription of either azithromycin or clarithromycin, amoxicillin/clavulanic acid or doxycycline after initial amoxicillin are all plausible changes in treatment strategy.

Our data indicate that fosfomycin and ciprofloxacin are increasingly used as second drug in an antibiotic treatment course. This could be the result of high switching from trimethoprim and nitrofurantoin and the increase in cystitis episodes [18]. Treatment failure due to resistance to first choice antibiotics could also be (partly) responsible for this observation, however, as we do not know the reason for a switch firm conclusions cannot be drawn. The increase in fosfomycin being used as a second agent might be the result of changes in the Dutch guidelines for urinary tract infections. Since 2005 fosfomycin is considered the second option to treat cystitis, making it plausible that fosfomycin use (as first and second prescriptions) increases in the following years.

### Strengths and limitations of the study

The main strength of the study is the use of a nationwide database containing over 90% of total dispensed antibiotics. The use of a database containing pharmacy dispensing data instead of prescription data results in more valid estimations of consumed antibiotics. Because patients in the Netherlands are often linked to one pharmacy, it is possible to monitor subsequent antibiotic use based on pharmacy data. A recent study showed that only 2% of patients regularly switch between pharmacies [19]. In contrast to earlier antibiotic switch studies that report data on specific diagnoses, or specific groups of antibiotics [11–13], our study gives an overview of the total outpatient antibiotic use. The greatest value of our approach is the analysis and visualisation of consecutive antibiotic use and distinguishing between prolongations and switches. In contrast to other studies that define switches as a prescription of a new antibiotic within 1 month after index date [11–13], our study uses a calculated end date of the first antibiotic to define antibiotic switches. The chance of including treatment for new or unrelated infections instead of prolonged treatment of the initial one is largely reduced by this method. Furthermore, we excluded patients on (nearly) chronic antibiotics because these could distort the data. In these patients antibiotics are mainly used for the prevention of recurrent infections instead of treating acute infectious disease episodes.

However, there are also limitations to our study that need to be addressed. First, the indication for prescription was not available. With the indication it would have been possible to specifically identify those switches that deviate from national treatment guidelines. Second, the data only contains outpatient pharmacy data. Data on treatment failures leading to hospital admission, visits to the emergency department, or antibiotics previously used in the hospital setting were not available and could not be incorporated. Finally, we were not able to determine the actual reason for the antibiotic switches. Although we speculate that an antibiotic switch reflects ineffectiveness of the treatment, possibly as a result of bacterial resistance, there can be several other explanations. Switches can also be a result of doctors’ behaviour (wrong diagnosis, inappropriate prescribing), patients’ behaviour (non-compliance, inappropriate expectations and re-counselling), or progression of the infection itself (including complications), or availability of the results of in vitro antimicrobial susceptibility testing. The evidence linking antibiotic treatment failure and antibiotic resistance is considered to be weak [20, 21]. Therefore, we cannot expect that bacterial resistance is the major cause of treatment failure and antibiotic switches.

### Future challenges

The dataset could be enriched by linking pharmacy data with clinical patient data, the antibiotic indication, outcomes of antimicrobial susceptibility tests, clinical outcomes and complications. However, there are security, privacy and technical issues that need to be addressed before such linking can be performed. Another challenge is to study seasonal and demographic differences in switch percentages for antibiotics. For European comparison studies, we encourage others to also evaluate trends in consecutive antibiotic use and switching patterns. These issues can provide even more detailed information that can help policymaking.

## Conclusion

The vast majority of antibiotics are prescribed as single courses, also antibiotics that are generally considered as 2nd- or 3rd-choice treatment. This is not in line with current guidelines. Switch percentages of antibiotics are low with an average percentage of 3%. The highest switch percentages were found for trimethoprim and nitrofurantoin. The most common antibiotic switches are in line with guideline recommendations. The data contributes to the knowledge regarding antibiotic switch patterns in the outpatient setting.

## Abbreviations

ATC: Anatomical therapeutic chemical classification
DDD: Defined daily dose
SFK: Foundation for Pharmaceutical Statistics
